# Andrographolide and Fucoidan Induce a Synergistic Antiviral Response In Vitro Against Infectious Pancreatic Necrosis Virus

**DOI:** 10.3390/molecules30112443

**Published:** 2025-06-03

**Authors:** Mateus Frazao, Daniela Espinoza, Sergio Canales-Muñoz, Catalina Millán-Hidalgo, Benjamín Ulloa-Sarmiento, Ivana Orellana, J. Andrés Rivas-Pardo, Mónica Imarai, Eva Vallejos-Vidal, Felipe E. Reyes-López, Daniela Toro-Ascuy, Sebastián Reyes-Cerpa

**Affiliations:** 1 Centro de Genómica y Bioinformática, Facultad de Ciencias, Ingeniería y Tecnología, Universidad Mayor, Santiago 8580745, Chile; mateus.frazao@mayor.cl (M.F.); sergio.canalesm@mayor.cl (S.C.-M.); benjamin.ulloas@mayor.cl (B.U.-S.); ivana.orellana01@mayor.cl (I.O.); jaime.rivas@umayor.cl (J.A.R.-P.); 2 Centro de Biotecnología Acuícola, Departamento de Biología, Facultad de Química y Biología, Universidad de Santiago de Chile, Santiago 9170002, Chile; daniela.espinoza@usach.cl (D.E.); monica.imarai@usach.cl (M.I.); eva.vallejosv@usach.cl (E.V.-V.); felipe.reyes.l@usach.cl (F.E.R.-L.); 3 Escuela de Biotecnología, Facultad de Ciencias, Ingeniería y Tecnología, Universidad Mayor, Santiago 8580745, Chile; catalina.millan@mayor.cl; 4 Laboratorio de Virología, Departamento de Biología, Facultad de Ciencias, Universidad de Chile, Santiago 8380000, Chile; 5 Núcleo de Investigación en Producción y Salud de Especies Acuáticas (NIP-SEA), Facultad de Medicina Veterinaria y Agronomía, Universidad de Las Américas, La Florida, Santiago 8242125, Chile; 6 Centro de Nanociencia y Nanotecnología CEDENNA, Universidad de Santiago de Chile, Santiago 9170002, Chile

**Keywords:** andrographolide, fucoidan, antiviral response, infectious pancreatic necrosis virus, Atlantic salmon macrophages

## Abstract

Andrographolide, fucoidan, or a combination of both compounds were evaluated to determine their effects on the antiviral response in the Atlantic salmon macrophage-like cell line (SHK-1) infected with infectious pancreatic necrosis virus (IPNV). We assessed the transcript expression levels of key molecules involved in the interferon (IFN)-dependent antiviral response, as well as the viral load in cells treated with these compounds. In non-infected cells, incubation with either fucoidan, andrographolide, or a mixture of both resulted in an increase in the transcript expression of IFNα1 and various interferon-stimulated genes (ISGs). In IPNV-infected cells, treatment with either fucoidan or andrographolide separately did not significantly enhance the antiviral response compared to that of infected cells that had not previously been treated with these compounds. In contrast, the combination of andrographolide and fucoidan led to a marked increase in the transcript expression of viperin and a significant reduction in viral load. Overall, combining andrographolide and fucoidan resulted in a greater reduction in IPNV viral load in infected cells than that noted when the compounds were administered individually. Our findings suggest that pre-incubation with this mixture promotes the establishment of a protective antiviral state against IPNV, likely mediated by an IFN-dependent response.

## 1. Introduction

The international community faces a significant challenge in feeding 9.7 billion people by 2050, aiming to eradicate hunger, food insecurity, and malnutrition by this date. This challenge is exacerbated by climate change, water scarcity, pollution, biodiversity loss, and other human-induced pressures [1]. Aquaculture, the fastest-growing sector in global animal food production, is becoming the primary source of aquatic food for human consumption. This industry is crucial for meeting the increasing global demand for seafood and is generally affordable for low-income populations, ensuring their access to nutritious options. Aquaculture provides a substantial source of protein and essential nutrients, such as omega-3 fatty acids, minerals, and vitamins [1,2]. Over the past 30 years, aquaculture has expanded rapidly [3]. Notably, Atlantic salmon (*Salmo salar*) production has surged, especially in Northern Europe and North and South America, with Norway and Chile leading the way as the top producers [1]. However, this rapid growth comes with side effects associated with high stocking densities, exposing fish to environmental and husbandry-related stresses that can negatively impact their welfare and performance. Additionally, increased disease susceptibility has been observed due to a reduced immune response, allowing pathogens to thrive more effectively [3,4,5].

Feed additives play a crucial role in maintaining fish health by stimulating the immune system, enhancing weight gain, improving feed efficiency, and increasing disease resistance in cultured fish [3]. Immunonutrition involves the use of ingredients or additives to modulate the immune response [6,7]. In aquatic animals, functional feed additives can stimulate the innate immune system or positively influence intestinal microbiota [8], thereby enhancing digestion while preventing the growth of harmful pathogens [6,9]. Numerous studies have reported the use of plants, herbs, algae, fungi extracts, and pathogen-associated molecular patterns (PAMPs) from bacteria and viruses as immunostimulants [3,5,6,9,10,11,12,13,14,15,16]. Seaweeds (macroalgae) are particularly noteworthy due to their diversity and the range of bioactive compounds they contain. They are classified into three groups based on pigments and phylogeny: green algae (Chlorophyta), red algae (Rhodophyta), and brown algae (Phaeophyta) [14]. Many types of seaweed have been utilized for centuries to nourish ruminant livestock, sheep, and pigs [14,17]. However, knowledge regarding their effects on the immune response of farmed animals, mainly fish, is still limited [14,18]. Fucoidan, a complex long-chain sulfated polysaccharide found in various brown algae species, such as *Fucus vesiculosus* and *Laminaria japonica*, is particularly noteworthy. As a bioactive molecule, fucoidan promotes immunostimulation, enhances phagocytic activity, improves antioxidant capabilities, and boosts growth and survival in shrimp and fish [16,18,19,20,21]. Medicinal herbs and plants have been recognized as immunostimulants for thousands of years and are increasingly being used in aquaculture as alternatives to antibiotics [22]. *Andrographis paniculata*, a plant in the Acanthaceae family, contains various bioactive compounds, with andrographolide being the most notable. Extracts from *A. paniculata* induce a non-specific immune response in fish and exhibit antibacterial activity [23,24].

Hernández et al. conducted a study on a dietary supplement called Futerpenol^®^, which contains fucoidans and labdane diterpenes as the main active compounds, deriving from an Acanthaceae family herb and different brown seaweeds [25]. They evaluated its effects in vitro using Atlantic salmon macrophages and in vivo in rainbow trout (*Oncorhynchus mykiss*) that were challenged with *Piscirickettsia salmonis*. Their findings showed an increased expression of type I IFN and interleukin-12 (IL-12) in vitro and reduced cellular cytotoxicity caused by *P. salmonis*. In rainbow trout infected with *P. salmonis* and fed Futerpenol^®^, they also noted an increase in survival rates [25]. Although the expression of type I IFN was enhanced, the specific antiviral effects of Futerpenol^®^ were not investigated. Futerpenol^®^ is a patented phytopharmaceutical that exhibits an antiviral effect against IPNV in Atlantic salmon cells infected with the virus. However, the mechanism explaining its effect has not been addressed [26].

The infectious pancreatic necrosis virus (IPNV) belongs to the Aquabirnavirus genus within the Birnaviridae family. IPNV is a non-enveloped, icosahedral, bisegmented dsRNA virus and is the etiological agent of Infectious pancreatic necrosis (IPN). This widespread disease leads to highly contagious systemic infections and high mortality rates among farmed salmonid species, resulting in significant economic losses [27,28,29]. Young salmonids are particularly susceptible to IPNV and exhibit symptoms such as erratic corkscrew swimming, exophthalmia (bulging eyes), darkened skin, pale gills, and distended abdomens. Internally, affected salmonids show signs of petechial hemorrhages in pancreatic tissue, hepatic necrosis, and intestinal mucosal necrosis, contributing to high mortality rates [27,28,29,30]. The virus can persist in surviving fish, with asymptomatic individuals shedding IPNV and acting as reservoirs, which helps establish endemic areas. The persistence of the infection is influenced by the balance between viral replication and the immune response, which often shows an imbalance in cytokine expression, including the downregulation of pro-inflammatory cytokines, upregulation of anti-inflammatory cytokine transcripts, and a limited induction of the antiviral cytokine IFNα [29,31,32,33].

Over the past decade, control strategies, such as vaccination and genetic management through quantitative trait locus (QTL) analysis, along with the selective breeding of resistant Atlantic salmon families, have helped reduce the incidence of IPNV infections. However, these measures are still insufficient due to the emergence of new IPNV variants that adversely affect genetically resistant farmed Atlantic salmon [30,34]. Consequently, there is an urgent need for new and alternative antiviral strategies against IPNV, with a focus on exploring naturally derived antivirals. This work evaluates the antiviral response induced by the combined use of andrographolide and fucoidan in an Atlantic salmon macrophage-like cell line (SHK-1) infected with IPNV.

## 2. Results

### 2.1. Quantification of Cytotoxicity Induced by Andrographolide, Fucoidan, or Their Mixture in SHK-1 Cells

**Figure 1:**
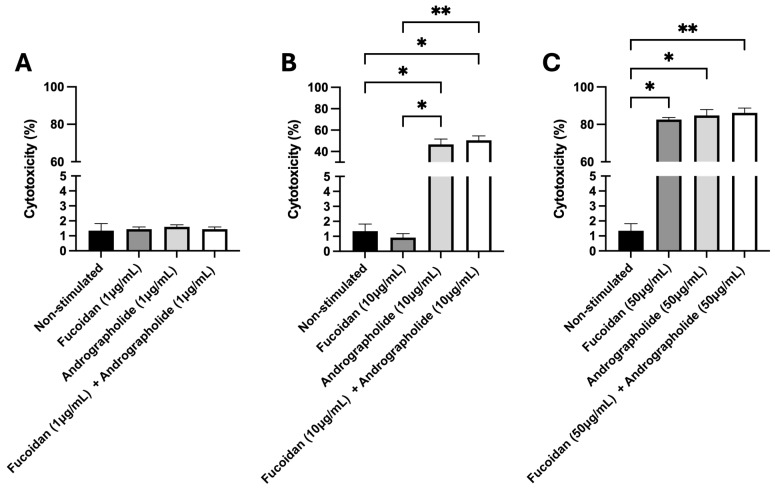
Evaluation of cytotoxicity induced by fucoidan, andrographolide, or their mixture. The cytotoxicity was assessed 5 days post-incubation with the compounds by measuring LDH enzymatic activity in the extracellular medium. (**A**) Cytotoxicity induced by fucoidan (1 µg/mL), andrographolide (1 µg/mL), or a mixture of andrographolide and fucoidan at 1 µg/mL each. (**B**) Cytotoxicity induced by fucoidan (10 µg/mL), andrographolide (10 µg/mL), or a mixture of andrographolide and fucoidan at 10 µg/mL each. (**C**) Cytotoxicity induced by fucoidan (50 µg/mL), andrographolide (50 µg/mL), or a mixture of andrographolide and fucoidan at 50 µg/mL each. Statistical analysis was conducted using non-parametric ANOVA, with Dunn’s multiple comparison test. Values are given as the mean ± standard error of the mean from three independent experiments. Asterisks denote statistical difference between stimulated (fucoidan, andrographolide, or a mixture of andrographolide/fucoidan) and non-stimulated SHK-1 cells, as follows: * *p* < 0.05; ** *p* < 0.01.

The cytotoxic effects of andrographolide, fucoidan, and their mixture were evaluated in SHK-1 cells using the LDH assay as a reporter for cell death (see Supplementary Figure S1A). The results showed that the percentage of cytotoxicity was around 1% in cells treated for 5 days with fucoidan (1 µg/mL), andrographolide (1 µg/mL), or a 1:1 mixture of fucoidan/andrographolide at 1 µg/mL each (Figure 1A). Fucoidan only induced cytotoxicity in SHK-1 cells at a 50 µg/mL concentration, with no cytotoxic effects observed at 10 µg/mL. In contrast, andrographolide exhibited cytotoxic effects at 10 µg/mL and 50 µg/mL. When the two compounds were combined in a 1:1 ratio, cytotoxicity was also observed at both 10 µg/mL and 50 µg/mL (Figure 1B,C). Therefore, for subsequent experiments, we will evaluate fucoidan, andrographolide, and their mixture at a final concentration of 1 µg/mL.

### 2.2. Induction of the Transcript Expression of IFNα1 and Interferon-Stimulated Genes (ISGs) by Andrographolide, Fucoidan, and Their Mixture

**Figure 2:**
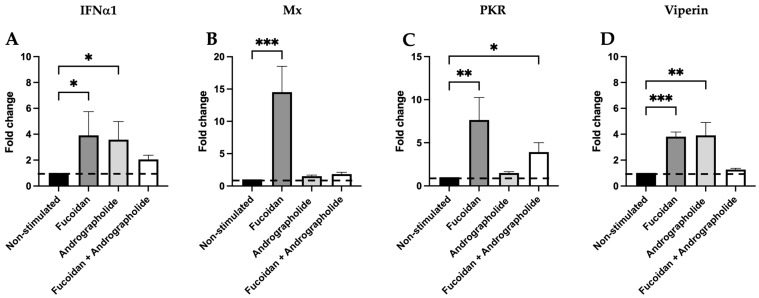
Antiviral cytokine gene expression in SHK-1 cells induced by fucoidan (1 µg/mL), andrographolide (1 µg/mL), or a mixture of andrographolide/fucoidan at 1 µg/mL each one for 24 h. The relative gene expression of (**A**) IFNα1, (**B**) Mx, (**C**) PKR, and (**D**) viperin was determined via the 2^−ΔΔCT^ method described by Rao et al., 2013 [35], with 18S rDNA transcript expression as a reference gene and non-stimulated SHK-1 cells as the control condition (black bars). The dashed line represents the baseline transcript expression in the non-stimulated cells. Statistical analysis was conducted using non-parametric ANOVA, with Dunn’s multiple comparison test. Values are given as the mean ± standard error of the mean from three independent experiments. Asterisks denote statistical differences between stimulated (fucoidan, andrographolide, or a mixture of andrographolide/fucoidan) and non-stimulated SHK-1 cells, as follows: * *p* < 0.05; ** *p* < 0.01; *** *p* < 0.001.

To investigate whether the transcript expression of IFNα1 and ISGs (Mx, PKR, and viperin) are induced by andrographolide, fucoidan, or a mixture of them, we incubated these compounds in SHK-1 cells for 24 h (Supplementary Figure S1B). The results showed that fucoidan induced IFNα1 transcript expression by 3.9-fold, while andrographolide induced it by 3.6-fold, compared to the IFNα1 transcript expression in non-stimulated SHK-1 cells. However, these increases were not observed in cells incubated with a mixture of andrographolide and fucoidan (Figure 2A).

The Mx transcript expression was only significantly induced in the SHK-1 cells incubated with fucoidan for 24 h, showing a 14.5-fold increase compared to the results for the non-stimulated cells (Figure 2B). PKR transcript expression was significatively induced by 7.6-fold with fucoidan and 3.9-fold with the mixture of fucoidan and andrographolide (Figure 2C). Similarly, viperin transcript expression was induced by 3.8-fold with fucoidan and 3.9-fold with andrographolide compared to the results for the non-stimulated cells. However, this increase in viperin expression was not observed in the presence of both compounds in a mixture (Figure 2D).

### 2.3. Synergistic Activity of Andrographolide/Fucoidan Mixture Against IPNV

To assess the effect of pre-treatment with each compound or their combination on the gene expression of antiviral cytokines in SHK-1 cells infected by IPNV, we incubated SHK-1 cells with fucoidan (1 µg/mL), andrographolide (1 µg/mL), or a mixture of both compounds at 1 µg/mL each for 24 h. Subsequently, the cells were infected with IPNV at MOI 0.5 PFUs/cell for 120 h (Supplementary Figure S2). As a control, SHK-1 cells were incubated with the andrographolide and fucoidan vehicles for 24 h before infecting them with IPNV.

**Figure 3:**
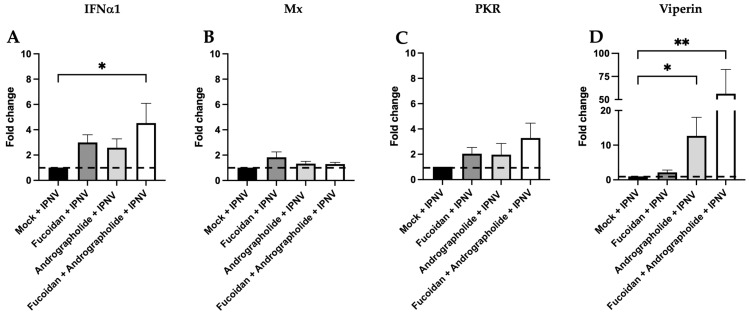
Antiviral cytokine gene expression in SHK-1 cells infected by IPNV for 120 h and previously pre-treated for 24 h with fucoidan (1 µg/mL), andrographolide (1 µg/mL), or a mixture of andrographolide and fucoidan at 1 µg/mL each. The relative gene expression of (**A**) IFNα1, (**B**) Mx, (**C**) PKR, and (**D**) viperin was determined by the 2^−ΔΔCT^ method described by Rao et al., 2013 [35], using 18S rDNA transcript expression as a reference gene. The control condition consisted of SHK-1 cells infected with IPNV for 120 h and pre-treated with the vehicles of andrographolide and fucoidan for 24 h (mock + IPNV; black bars). The dashed line indicates the baseline transcript expression level in the control condition (mock + IPNV). Statistical analysis was conducted using non-parametric ANOVA via Dunn’s multiple comparison test. Values are given as the mean ± standard error of the mean from three independent experiments. Asterisks denote statistical difference between stimulated (fucoidan, andrographolide, or a mixture of andrographolide/fucoidan) and non-stimulated SHK-1 cells, as follows: * *p* < 0.05; ** *p* < 0.01.

The transcript expression of IFNα1 was increased 4.5-fold, only when both andrographolide and fucoidan were combined in the IPNV-infected cells. However, when these compounds were applied separately, they did not induce any changes in the transcript expression of IFNa1 in the IPNV-infected cells (see Figure 3A). On the other hand, the expression levels of Mx and PKR remained unchanged in the presence of both compounds, whether applied separately or together, in the IPNV-infected cells (Figure 3C,D). Conversely, viperin transcript expression was induced by 12.7-fold and 56.4-fold in IPNV-infected cells that had been previously treated with andrographolide and with the mixture of andrographolide and fucoidan, respectively, compared to the results for the control group of IPNV-infected cells (control group) (Figure 3D).

### 2.4. Andrographolide/Fucoidan Mixture Exhibits Potent and Synergistic Antiviral Activity Against IPNV

**Figure 4:**
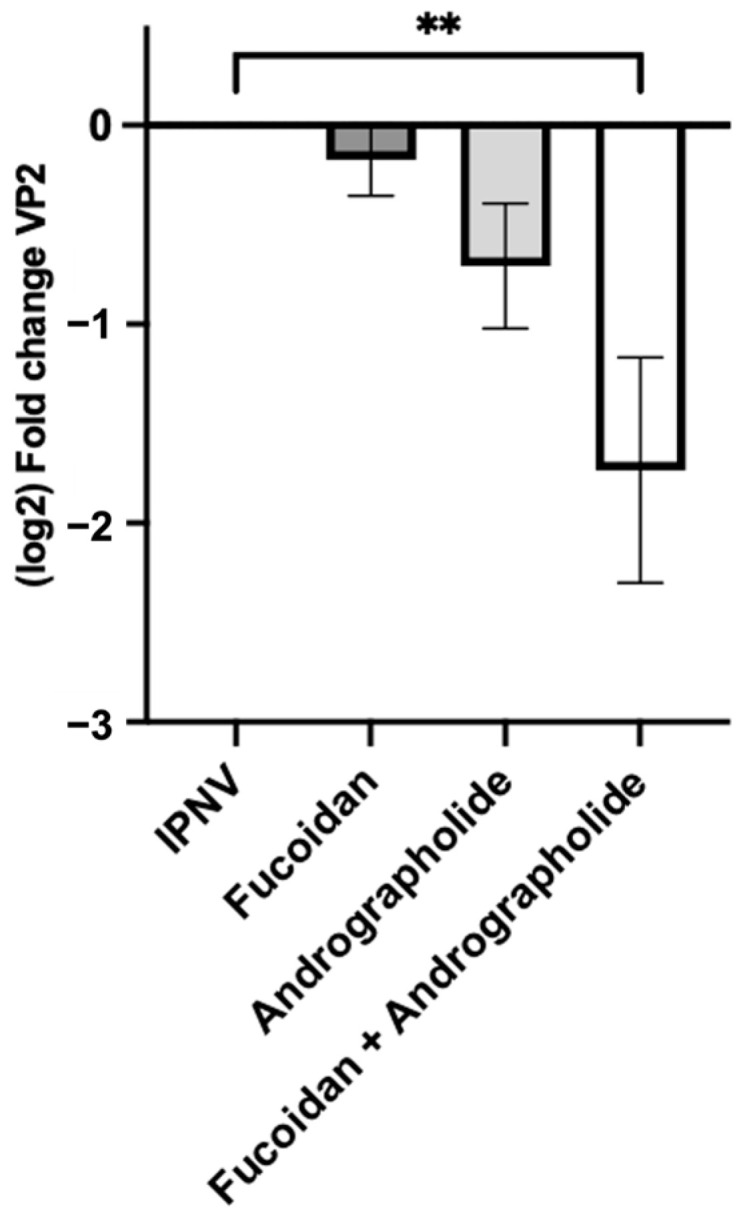
Quantification of viral load in supernatant of IPNV-infected cells. The number of copies of the VP2 gene was used to determine the viral load by absolute quantification via qPCR. The VP2 copies detected in the supernatant from infected SHK-1 cells previously treated with fucoidan, andrographolide, or a mixture of both were normalized as the fold change in regards to the number of copies of VP2 detected in the supernatant of infected cells that were not previously stimulated. Statistical analysis was conducted using non-parametric ANOVA, with Dunn’s multiple comparison test. Values are given as the mean ± standard error of the mean from three independent experiments. ** *p* < 0.01.

We used two approaches to assess the antiviral activity of the evaluated compounds or a mixture of them: (a) evaluation of viral load in the supernatant of the infected cells, and (b) determination of the number of infectious particles as plaque-forming units (PFUs). First, to evaluate the viral load, we incubated fucoidan (1 µg/mL), andrographolide (1 µg/mL), or a combination of both at 1 µg/mL each in SHK-1 cells for 24 h. After this, the cells were infected with IPNV at MOI 1 PFUs/cell for 120 h (Supplementary Figure S2). We assessed the viral load by quantifying the number of copies of the VP2 gene in the supernatant of the infected cells. Treatment with fucoidan and andrographolide alone resulted in a non-significant decrease in the viral load compared to that of the mock-incubated group. On the contrary, when evaluating the mixture of both compounds, a significant decrease was observed compared to that for the IPNV-infected cells that had not been pre-treated with the compounds (Figure 4).

**Figure 5:**
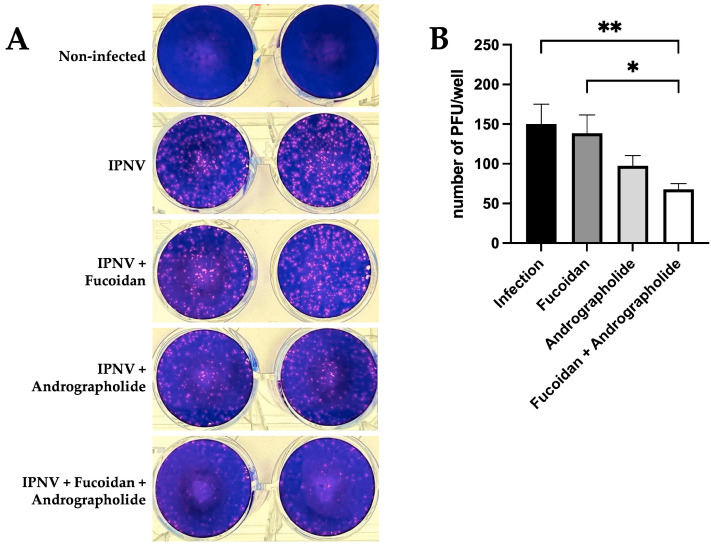
Antiviral effect of pre-treatment with andrographolide/fucoidan mixture in CHSE-214 cells infected by IPNV. The antiviral activity of fucoidan (1 µg/mL), andrographolide (1 µg/mL), or a mixture of andrographolide/fucoidan (1 µg/mL each one) against IPNV was assessed by plaque reduction assay in CHSE-214 cells. (**A**) Representative results of PFU-forming plate assay (the left and right wells represent technical duplicates); (**B**) quantification of PFUs/well obtained for each treatment. Statistical analysis was conducted using non-parametric ANOVA, with Dunn’s multiple comparison test. Values are given as the mean ± standard error of the mean from three independent experiments. * *p* < 0.05; ** *p* < 0.01.

Next, the PFUs were determined in the CHSE-214 cells that had been incubated for 24 h with the compounds or their mixture. For this purpose, IPNV at an MOI of 0.002 PFUs/cell was used for an adequate count of the number of PFUs obtained. We observed a reduction in the PFUs/well when infected cells were previously pre-treated with a mixture of andrographolide and fucoidan (Figure 5A). When quantifying the PFUs/well obtained for each treatment, we observed that incubation with fucoidan does not affect the number of infectious particles. In contrast, when quantifying the number of PFUs in cells treated with only andrographolide, a 30% reduction in infectious particles was observed, although this does not achieve statistical significance. However, when the cells were treated with a combination of andrographolide and fucoidan before infection with IPNV, there was an observed reduction of over 50% in the number of infectious particles compared to the results for infected cells that had not been previously stimulated (Figure 5B). As a control, we evaluated the number of PFUs/well in cells infected with IPNV and previously incubated with the andrographolide and fucoidan vehicles, but we did not observe a reduction in the number of infectious particles (Supplementary Figure S3).

## 3. Discussion

Developing an effective and long-lasting antiviral response to combat IPNV outbreaks in Atlantic salmon is of utmost importance. Reyes-López et al. described a differential expression patterns of immune-related genes associated with IPN susceptibility and resistance in Atlantic salmon. The authors observed a high upregulation of IFNα in susceptible families and a moderate increase in resistant families at day 1 post-infection. However, the IFNα expression dropped to basal values at day 5 post-infection, only in susceptible fish. In resistant families, the levels remained high or increased, suggesting that viral mechanisms of immune evasion may work in those fish. In contrast, a long-lasting IFN-induced antiviral state persists in resistant families, reflecting the importance of developing and maintaining a prolonged and constant response, mediated by IFNα, to induce a protective response against IPNV [36]. Although vaccination is recognized as the best method to prevent viral diseases and there are several vaccines available against IPNV, mainly inactivated vaccines, most of them are prepared against IPNV genogroup V, which cannot be appropriately applied to protect against other IPNV genogroups [37]. Recently, Li et al. evaluated the cross-protection of inactive vaccines developed with genogroups I and V. As a result, the authors described that the IPNV genogroup V vaccine provided cross-protection to the IPNV genogroup I strain. However, when fish are challenged with IPNV genogroup V, the IPNV genogroup I vaccine does not significantly reduce viral load after 120 days [38]. The absence of vaccines that provide efficient and universal protection against IPNV highlights the need to develop new tools and antiviral strategies that induce a strong and effective innate immune response [38,39].

Fucoidan is a polysaccharide that contains substantial percentages of L-fucose and sulfate ester groups, mainly derived from brown seaweed [40]. Fucoidans generally exhibit simple chemical compositions, mainly composed of fucose and sulfate (for example, from *Fucus vesiculosus*); many fucoidans display complex structures that may include additional monosaccharides (mannose, galactose, glucose, xylose, and others), uronic acids, and even acetyl groups and proteins [40]. Fucoidans from different species have been extensively studied due to their wide range of biological activities, including antiviral [40,41,42,43], anti-tumor [40,44,45,46], immunomodulatory, and anti-inflammatory properties [40,47,48]. Similarly, andrographolide, a diterpenoid labdane contained in *Andrographis paniculata* [49], has also shown a broad range of biological activities, such as antioxidant [50], antibacterial [49,51,52], antiviral [49,53], and anti-inflammatory effects [49,50,54].

During a viral infection, the virus must overcome cytoplasmic barriers associated with innate immune response, such as IFN-mediated immunity [55]. IFNs are key regulators of the immune response against viruses, including a family of pro-inflammatory, immunomodulatory, and pleiotropic cytokines that induce an antiviral state by upregulating hundreds of ISGs [55,56,57]. Choi et al. conducted a study analyzing the global transcriptomic changes in bone marrow-derived dendritic cells (BMDCs) from mice incubated with fucoidan for 24 h. The authors found that fucoidan activated pathogen recognition receptor (PRR) signaling, leading to type I IFN production and signaling, as well as the production of ISGs, e.g., 435 out of 950 upregulated genes were ISGs. This highlights a potential mechanism through which fucoidan exerts its antiviral activity [58]. Additionally, fucoidan has been show to inhibit hepatitis B virus (HBV) replication by activating the antiviral immune response via the mitogen-activated protein kinases (MAPKs) extracellular signal-regulated kinase 1/2 (ERK1/2) pathway and subsequently enhancing the production of IFNα [59]. Fucoidan exhibits a broad spectrum of antiviral activity; in addition to promoting an IFN-dependent antiviral response, it can act directly against viruses by interfering with their replicative cycle. It can prevent viral entry into cells by interacting with enveloped viral particles, cell receptors, or viral enzymes, thereby inhibiting viral adsorption and the transmission of the virus from cell to cell [60,61]. Several studies suggest that polysaccharides possess antiviral activity linked to their anionic groups, especially sulfate groups, which interact with viral proteins and disrupt viral replication [41,60,62,63,64,65].

In our research, we found that stimulation with fucoidan alone for 24 h induces the expression of IPNV and the evaluated ISGs (PKR, Mx, and viperin), which is expected, according the discussion above. However, fucoidan does not elicit an efficient and protective antiviral response in cells that are infected with the virus and have been pre-treated with fucoidan. The expression of the antiviral response transcripts remains unchanged when compared to the transcript expression in cells infected with IPNV and not previously treated with fucoidan. Likewise, we did not observe a decrease in the viral load of IPNV in the infected cells, according to the detection of viral genomic RNA in the infection supernatant, as noted in the plaque reduction assay. This lack of an effective antiviral response may be attributed to the immune response suppression mechanisms induced by IPNV, which allow for the establishment of a persistent infection, where an upregulation of anti-inflammatory cytokines, a downregulation of other pro-inflammatory cytokines, and even an increase in viral proteins that antagonize IFNa1 promoter activation and inhibit IFN-signaling has been described [29,31,66,67,68]. Additionally, the ineffectiveness of fucoidan alone in mounting an antiviral response in IPNV-infected cells may be related to the concentration of polysaccharide used. For instance, Li et al. observed that fucoidan enhanced the phagocytic capacity of *M. amblycephala* macrophages and the transcript expression of *CXCL8* and *IL-1β* at a final concentration of 100 μg/mL [69]. On the other hand, Caipang et al. observed that fucoidan stimulated the immune response of head kidney leukocytes from Atlantic cod at a final concentration of 10 μg/mL, inducing the cellular myeloperoxidase activity, which could be an important factor in the bacterial immune response [70]. In our results, concentrations of 50 μg/mL, but not 10 μg/mL, of fucoidan were cytotoxic in SHK-1 cells incubated for 5 days.

Andrographolide has been suggested as an immunostimulant that, in mice, can increase the cell-mediated immune responses of natural killer cells, the antibody-dependent cellular cytotoxicity (ADCC), and the antibody-dependent complement-mediated cytotoxicity (ACC). Moreover, andrographolide has been suggested as stimulating cytotoxic T lymphocyte (CTL) production and IL-2 and IFNγ production by T cells [71,72]. Several studies suggest that andrographolide induces the host’s innate antiviral response, activating the expression of PKR, RIG-I, and IFNα [73]. Similarly, in the human hepatoma cell line infected by the hepatitis C virus (HCV), andrographolide induces an antiviral response via heme oxygenase-1 (HO-1) induction, increasing IFNα expression and the inhibition of HCV NS3/4A protease activity [74]. Similarly, andrographolide suppresses RSV replication via HO-1 induction in human airway epithelial cells but does not activate the antiviral IFN response [75]. On the other hand, andrographolide has been described to inhibit influenza virus replication; it may do so in a direct manner by binding to HA and NA to prevent the virus from entering or leaving the host cell; it also decreases inflammation by suppressing pro-inflammatory cytokines and chemokines via inhibiting the NF-κB signaling pathway, and even downregulating the Janus kinase/signal transducer and transcription (JAK/STAT) activation signals [49]. Given the above, andrographolide would inhibit the replication of several viruses through multiple pathways.

In our study, incubation of SHK-1 cells with andrographolide alone for 24 h induces the expression of IFNα and viperin transcripts. This effect was also observed in SHK-1 cells infected with IPNV and previously treated with andrographolide. Although the changes were not statistically significant, there appeared to be a reduction in the viral load of IPNV in the infection supernatant, as well as a decrease in the number of infectious virus particles. This slight antiviral effect observed only for the incubation with andrographolide in SHK-1 cells infected by IPNV could be attributed to the concentrations used in our experiments. Wang et al. reported immunomodulatory effects on murine macrophage polarization at 10 μg/mL [76]. On the other hand, Chaopreecha et al. observed an antiviral effect against SARS-CoV-2 for andrographolide at 11 μM [77], a concentration like the one we use in our work. Similarly, Gupta et al. reported an antiviral effect against chikungunya virus infection by upregulating the protein expression of IFNα, PKR, IRF3, IRF7, and RIG-I from 0.5 μg/mL of andrographolide in human PBMCs and THP-1 cells [78]. Our results showed that 10 μg/mL of andrographolide incubation for 5 days was cytotoxic in SHK-1 cells. Interestingly, when SHK-1 cells were incubated with a combination of fucoidan and andrographolide for 24 h, there was no significant increase in the expression of the evaluated antiviral transcripts. However, when SHK-1 cells infected with IPNV were pre-treated with this mixture, a notable increase in the expression of IFNα and viperin transcripts was observed 144 h after initial stimulation. These results suggest that the combination of fucoidan and andrographolide may induce an effective and synergistic antiviral response, as indicated by the decrease in viral load in the infection supernatants and the reduction in infectious viral particles seen in the lysis plaque assay.

Viperin, also known as cig5 and RSAD2, is a highly species-conserved, 361-aminoacid protein with a molecular mass of 42 kDa, which is expressed by a wide variety of mammals, reptiles, and fish [79]. Viperin is expressed in most cell types at very low basal levels. It has been demonstrated that viperin belongs to the ISG family and is induced by a type I IFN-dependent pathway. However, viperin can also be induced in a type I IFN-independent pathway mediated by IRF1, in addition to IRF3, activation, by peroxisomal MAVS responsible for the immediate expression of ISGs before type I IFN induction, resulting in a rapid antiviral effect [80]. Viperin is primarily anchored into the endoplasmic reticulum (ER), with the C-terminal part of the protein protruding into the cytosol, but it is also localized on lipid droplets (LD), which are involved in lipid storage and transport, as well as protein storage and degradation [80]. As for the antiviral effects of viperin, Rivera-Serrano et al. have categorized its actions into four main groups: (a) inhibition of viral RNA replication, (b) perturbation of the secretory pathway, (c) direct binding to viral proteins, and (d) dysregulation of lipid raft formation by altering lipid metabolism [81].

The science behind the mechanism of action of the combined use of labdane diterpenes and fucoidan against viral infections has not been adequately evaluated. Futerpenol^®^, a patented phytopharmaceutical utilized in aquaculture, features fucoidans and labdane diterpenes as its primary active compounds, and it has been shown to exert an antiviral effect against IPNV [26]. However, the mechanisms involved have not been addressed. Prior reports have shown that labdane diterpenes and fucoidan are effective against bacterial infections. For instance, Hernández et al. reported a protective cellular response in SHK-1 cells infected with a virulent strain of *P. salmonis*, mediated by the induction of IL-12 and IFNγ in those cells. In an in vivo challenge, the authors observed a higher survival rate in *P. salmonis*-infected fish when both compounds were included in their diet compared to the results for those fed a control diet [25]. While there are studies examining the antiviral effects of labdane diterpenes, like andrographolide, and sulfated polysaccharides, such as fucoidan, against various viral infections in different animal and fish species, this is the first study to explore the antiviral response initiated by their combined use against IPNV in Atlantic salmon. The combination of these compounds exhibited a synergistic antiviral effect, as their joint application resulted in better outcomes than those noted when each compound was used individually, even at lower concentrations. Despite these promising findings, further research is necessary to clarify the antiviral response induced by both compounds and to understand the innate immune response involved in reducing viral loads in infected cells.

## 4. Materials and Methods

### 4.1. Cell Culture

The SHK-1 cell line (*Salmo salar*, ECACC 97111106, European Collection of Authenticated Cell Cultures) was employed as the macrophage model [82], which has been previously used to evaluate Atlantic salmon macrophage–host interactions against IPNV [83,84,85]. SHK-1 cells were cultured at 16 °C in Leibovitz’s 15 medium (L-15, Corning, NY, USA) supplemented with 5% (*v*/*v*) fetal bovine serum (FBS) (Corning), 4 mM L-glutamine (Corning), 1% (*v*/*v*) 2-mercaptoethanol (2-ME, Gibco, Miami, FL, USA), 1× penicillin/streptomycin 100× (Corning), and 2.5 mg/mL Amphotericin-B (Corning).

The embryo-derived cells CHSE-214 (*Oncorhynchus tshawytscha*, ATCC CRL-1681, USA) were grown at 16 °C in Eagle’s minimum essential medium (MEM, Corning) supplemented with 10% (*v*/*v*) FBS (Corning), 10 mM HEPES (Corning), 1% (*v*/*v*) non-essential amino acids (Corning), 2 mM L-glutamine (Corning), and 50 μg/mL of gentamicin.

### 4.2. Propagation and Titration of the IPN Virus

The IPNV Dry Mills (DM) strain was propagated by inoculating CHSE-214 cells at a multiplicity of infection (MOI) of 5 PFUs/cell in minimum essential medium (MEM, Gibco, Grand Island, NY, USA) supplemented with 2% FBS, 2 mM L-glutamine (Gibco), and 100 UI/mL/100 mg/mL penicillin/streptomycin (Gibco), as previously described by Espinoza et al. 2024 [30]. Infected cultures were incubated at 16 °C until the cytopathic effect became evident. The viral inocula was titrated using the plaque lysis assay method [30,31,83,86].

### 4.3. Evaluation of the Cytotoxicity Induced by Andrographolide, Fucoidan, and a Mixture of Andrographolide and Fucoidan

Andrographolide (Sigma-Aldrich, Saint Louis, MO, USA) was prepared by dissolving in chloroform-methanol (1:1) at a concentration of 1 mg/mL, which was then diluted in L-15 medium to reach a final concentration of 1, 10, and 50 µg/mL. Fucoidan (Sigma-Aldrich) was prepared directly in L-15 medium at a final concentration of 1, 10, and 50 µg/mL. A mixture of andrographolide and fucoidan in a 1:1 ratio was also prepared, with each at a final concentration of 1, 10, and 50 µg/mL.

To assess the cytotoxicity of the evaluated compounds on SHK-1 cells, we measured the release of lactate dehydrogenase (LDH) into the extracellular medium, as previously described by Velasquez et al., 2024 [87] (Supplementary Figure S1A). SHK-1 cells were seeded at 15,000 cells/well in 96-well flat-bottom plates. After seeding, the cells were incubated with fucoidan (1, 10, and 50 µg/mL), andrographolide (1, 10, and 50 µg/mL), and a mixture of 1:1 of both compounds at 1, 10, and 50 µg/mL each (mixture). The vehicle control was prepared with the andrographolide vehicle (chloroform–methanol 1:1 at 0.1% (*v*/*v*)) and with the fucoidan vehicle (L-15 medium). The cytotoxicity was assessed in the SHK-1 cell culture at 5 days post-incubation using the Pierce LDH Cytotoxicity Assay Kit (Thermo Scientific, Waltham, MA, USA), following the manufacturer’s instructions.

### 4.4. Evaluation of Antiviral Transcripts Expression Induced by Andrographolide, Fucoidan, or Their Mixture in SHK-1 Cells

SHK 1 cells were seeded at 250,000 cells/well in 6-well flat bottom plates. Then, the cells were incubated with fucoidan (1 µg/mL), andrographolide (1 µg/mL), or a mixture of andrographolide and fucoidan at 1 µg/mL each (mixture). The andrographolide vehicle (chloroform–methanol 1:1 at 0.1% (*v*/*v*) in L-15 medium) and the fucoidan vehicle (L-15 medium) were used as vehicle controls. Incubation was carried out for 24 h (Supplementary Figure S1B).

RNA extraction from the stimulated cells was performed using TRIzol Reagent (Life Technologies, Waltham, MA, USA), according to the manufacturer’s instructions. Total RNA extracted was resuspended in molecular biology grade water (Corning), and the samples were incubated at 58 °C for 10 min. The total RNA concentration was quantified spectrometrically at 260 nm using a Nanoquant Infinite M200 pro (TECAN, Grödig, Austria) device. The purity of the sample was measured via the 260/280 nm wavelength ratio. Total RNA (1.5 μg) was treated with RQ1 RNase-free DNase (Promega, Madison, WI, USA), and cDNA synthesis was performed using reverse transcriptase M-MLV (Promega) and Oligo dT (Promega), following the manufacturer’s recommendations. The cDNA samples were stored until use at −20 °C.

Real-time PCR was performed in a QuantStudio™ 3 Real-Time PCR system (ThermoFisher) using Takyon™ ROX SYBR^®^ MasterMix blue dTTP kit (Eurogentec, Seraing, Belgium), according to the manufacturer’s instructions. PCR reactions were run in triplicate using a final concentration of primers of 0.2 µM and 2 µL of cDNA. The qPCR parameters were 50 °C for 2 min and 95 °C for 2 min, followed by 40 cycles at 95 °C for 15 s, 58 °C for 15 s, and 72 °C for 15 s. The primer sequences for the different genes are listed in Table 1. Amplification data were analyzed using the Design & Analysis Software 2.6.0 program. Relative gene expression analysis was carried out according to 2^−ΔΔCT^, as described by Rao et al., 2013 [35] using 18S rDNA transcript expression as a housekeeping gene. The results are expressed as the fold change in gene expression relative to that observed in the SHK-1 cells incubated only with the respective vehicles (non-stimulated).

### 4.5. Evaluation of Antiviral Transcripts Expression in SHK-1 Cells Infected by IPNV Pre-Treated with Andrographolide, Fucoidan, or Their Mixture

In order to determine the antiviral transcript expression in SHK-1 cells infected by IPNV and pre-treated with andrographolide, fucoidan, or their mixture, SHK-1 cells were seeded at 250,000 cells/well in 6-well flat bottom plates. Then, the cells were incubated with fucoidan (1 µg/mL), andrographolide (1 µg/mL), or a mixture of andrographolide and fucoidan at 1 µg/mL each (mixture) for 24 h at 16 °C. The andrographolide vehicle (chloroform-methanol 1:1 at 0.1% (*v*/*v*) in L-15 medium) and the fucoidan vehicle (L-15 medium) were used as vehicle controls. After stimulation, the cells were washed twice with PBS 1×, and the medium was replaced by L-15 medium supplemented with 2% FBS, 100 UI/mL/100 mg/mL penicillin/streptomycin (Gibco), and 2 mM L-glutamine (Gibco). Then, the SHK-1 cells were infected by IPNV (MOI: 0.5 PFUs/cell) for 1 h. After viral adsorption, the virus was removed by washing with PBS 1× three times, and the cells were incubated at 16 °C for 120 h with fresh L-15 supplemented medium (Supplementary Figure S2A).

Total RNA extraction, DNAse treatment, cDNA synthesis, and RT-qPCR analysis were performed following the above protocols. The results are shown as the fold change in gene expression relative to that observed in the SHK-1 cells infected with IPNV and pre-treated with the respective vehicles (IPNV).

### 4.6. Quantification of IPNV Viral Protein 2 (VP2) Gene by Quantitative PCR (qPCR) in SHK-1 Cells Pre-Treated with Andrographolide, Fucoidan, or Their Mixture

The number of copies of the VP2 gene was measured to evaluate the effect of pre-treatment of the evaluated compounds or their mixture on the viral load of IPNV released from the infected cells (Supplementary Figure S2A). SHK-1 cells were seeded at 250,000 cells/well in 6-well flat bottom plates. The cells were then incubated with fucoidan (1 µg/mL), andrographolide (1 µg/mL), or a mixture of andrographolide/fucoidan at 1 µg/mL each (mixture) for 24 h at 16 °C. The SHK-1 cells were incubated with the fucoidan vehicle or the andrographolide vehicle as controls. Following the pre-treatment, the cells were infected with IPNV at 1 PFU/cell. After 120 h of infection, viral RNA was extracted from the supernatant of the infected cells using the “Quick-RNA™ Viral Kit” from Zymo Research (Irvine, CA, USA), according to the manufacturer’s instructions. For the quantitative analysis, a One-Step qPCR was performed using the Takyon™ One-Step Kit Converter and the Takyon™ ROX SYBR^®^ MasterMix blue dTTP kit (Eurogentec). The qPCR reactions were run in triplicate, using a final concentration of primers specific for VP2 from IPNV of 0.2 µM (primers sequence listed in Table 1) and 4 µL of the extracted viral RNA. The amplification program used was the same as that used for the previous molecules. For VP2 amplification, primer alignment and reverse transcription were performed at 60 °C.

To achieve absolute quantification of the VP2 gene copies, a standard curve was established. The PCR amplification product of VP2 was cloned into a pGEM^®^-T Easy Vector System, following the manufacturer’s protocols. Serial dilutions ranging from 10^8^ to 10^1^ copies of plasmid were used to create the qPCR standard curve for VP2. The qPCR reaction was run alongside the serial dilutions of the plasmid, and the viral load of the samples was determined by interpolation from the standard curve using the cycle threshold (Ct) values obtained for each sample.

### 4.7. Determination of the Antiviral Activity of Fucoidan and Andrographolide Pre-Treatment via Plaque Reduction Assay

The CHSE-214 cells were seeded in 12-well plates and cultured until reaching 90% confluence in MEM medium supplemented with 2% FBS, 100 UI/mL/100 mg/mL penicillin/streptomycin (Gibco) and 2 mM L-glutamine (Gibco). The cells were treated with fucoidan (1 µg/mL), andrographolide (1 µg/mL), and the combination of these compounds for 24 h in MEM medium supplemented with 2% FBS at 18 °C. Cells treated with the andrographolide vehicle (chloroform–methanol 1:1 at 0.1% (*v*/*v*)) and the fucoidan vehicle were used as controls. After removal of the medium with the compounds and washing with 1X PBS, the cells were infected with IPNV (MOI 0.002 PFUs/cell) to obtain countable plaques, using uninfected CHSE-214 cells as the control. After 1 h of adsorption, the virus was removed, and the cells were incubated at 18 °C for 48 h in a semi-solid medium containing MEM supplemented with 10% FBS and 0.5% low-melting agarose (Gibco). Subsequently, the cells were fixed with 1 mL of 37% (*v*/*v*) formaldehyde (Winkler, Stuttgart, Germany) and incubated for 1 h. The agarose was removed, and 500 µL of 1% (*v*/*v*) crystal violet (Merck, Billerica, MA, USA) was added to each well to detect viral plaques. After 30 min of incubation, the excess crystal violet was removed, and the lysis plaques were quantified. The percentage reduction in PFUs for each treatment was compared with the results for the untreated infected cells (Supplementary Figure S2B).

The PFU counting was carried out using Image J 1.52g software. Briefly, In the “Image options”, colors were generated from white to black using the “Threshold” function. In cases of oversaturation, adjustments were made to ensure that the black spots of the lysis plaques were visible. Next, the “Process” tool was used to convert the image into a binary format with “Watershed”, allowing the program to differentiate and separate two lysis plaques that might be close together. Then, using the “Analyze” tool, the number of lysis plaques in each well was counted with the “Analyze Particles” option, setting the particle size from 0 to infinity, the circularity from 0 to 1, and selecting the “Show outlines” and “Display results” options.

### 4.8. Statistical Analysis

We used the GraphPad Prism^®^ 10 program (GraphPad Software, Inc., La Jolla, CA, USA) to calculate the mean and the standard error of the mean (SEM) and to create graphs and perform statistical tests. Data were statistically analyzed using non-parametric one-way analysis of variance (ANOVA), with Dunn’s multiple comparisons test. The *p* values < 0.05 were accepted as significant.

## 5. Conclusions

The lack of effective treatments for IPNV remains a significant concern. Natural compounds derived from plants and algae extracts may provide potent antiviral agents comparable to synthetic drugs. In our study, a combination of andrographolide and fucoidan applied before infection with IPNV in Atlantic salmon macrophages exhibited an antiviral effect that surpassed the effects of each compound used separately. This combination induces the expression of IFNα and viperin transcripts, thereby creating an antiviral state that is resistant to IPNV, suggesting that the mixture of these compounds could be developed as an antiviral agent. However, the mechanisms underlying the induction of this antiviral state in a more physiologically relevant model require further investigation.

## Figures and Tables

**Table 1:** Primers used in real-time PCR for gene expression analysis.

Gene	Sequence 5′→3′	GenBank Accession No.	Reference
PKR	CCCTCCTGTCCGAGCAGTTA	EF523422	This study
	AGCCTCCTTCTTCGTGTTCC		
Mx	CGATGCCCTCTCGAGCTGAA	NM_001139918	This study
	TGAGTGTGAGGTCTGGGACG		
Viperin	CTGTACGCTGGAAGGTGTTC	NM_001140939	This study
	GCCAACATCAAGGATGGACTT		
IFNα1	GGACAAGAAAAACCTGGACG	AY216594	[31]
	CTTTCCTGATGAGCTCCCAC		
VP2	GACCAAGTTCGACTTCCAGC	FN257531	[31]
	ATCGGCTTGGTGATGTTCTC		
18S	CCTTAGATGTCCGGGGCT	AJ427629	[36]
	CTCGGCGAAGGGTAGACA		

## Data Availability

The original contributions presented in this study are included in the article/Supplementary Material. Further inquiries can be directed to the corresponding authors.

## Supplementary Materials

The following Supporting Information can be downloaded at: https://www.mdpi.com/article/10.3390/molecules30112443/s1. Figure S1: Schematic illustration of the experimental design. Figure S2: Schematic illustration of the experimental design used to evaluate antiviral response in cells pre-treated with andrographolide, fucoidan, and their combination. Figure S3: The PFU-forming plate assay was visualized on CHSE-214 cells infected with IPNV, which were previously incubated with andrographolide and fucoidan vehicles.
