# Supplementary material for: Andrographolide and Fucoidan Induce a Synergistic Antiviral Response In Vitro Against Infectious Pancreatic Necrosis Virus
Source: Molecules. 2025 Jun 3;30(11):2443. doi: 10.3390/molecules30112443 (PMC12156450; doi:10.3390/molecules30112443)
Supplement: Supplementary file 1 [file molecules-30-02443-s001.zip › molecules-3597226-supplementary.pdf]

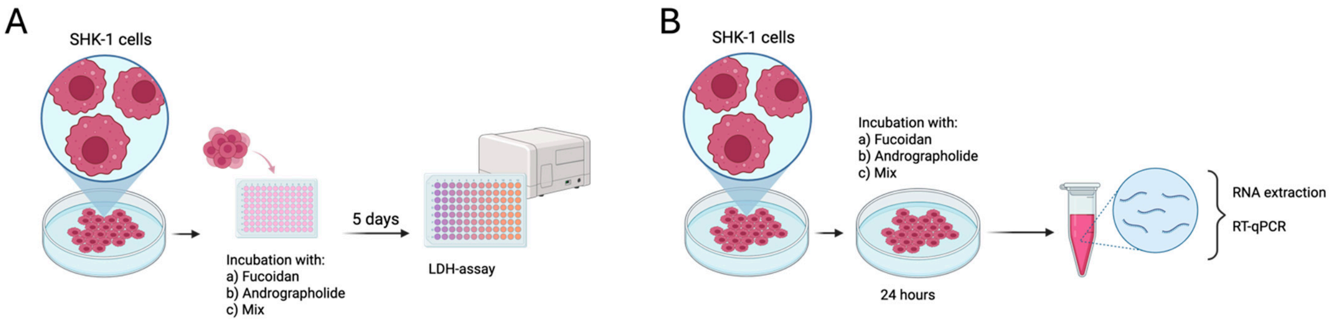

Supplementary Figure S1: Schematic illustration of the experimental design. A) Cytotoxicity induced by andrographolide, fucoidan, and their combination was evaluated in SHK-1 cells using the LDH assay to measure cell death. B) The experimental design for analyzing the transcript expression of type I IFN and ISGs (PKR, Mx, and viperin) in SHK-1 cells treated with andrographolide, fucoidan, and their combination. The cartoon illustration was created using BioRender.

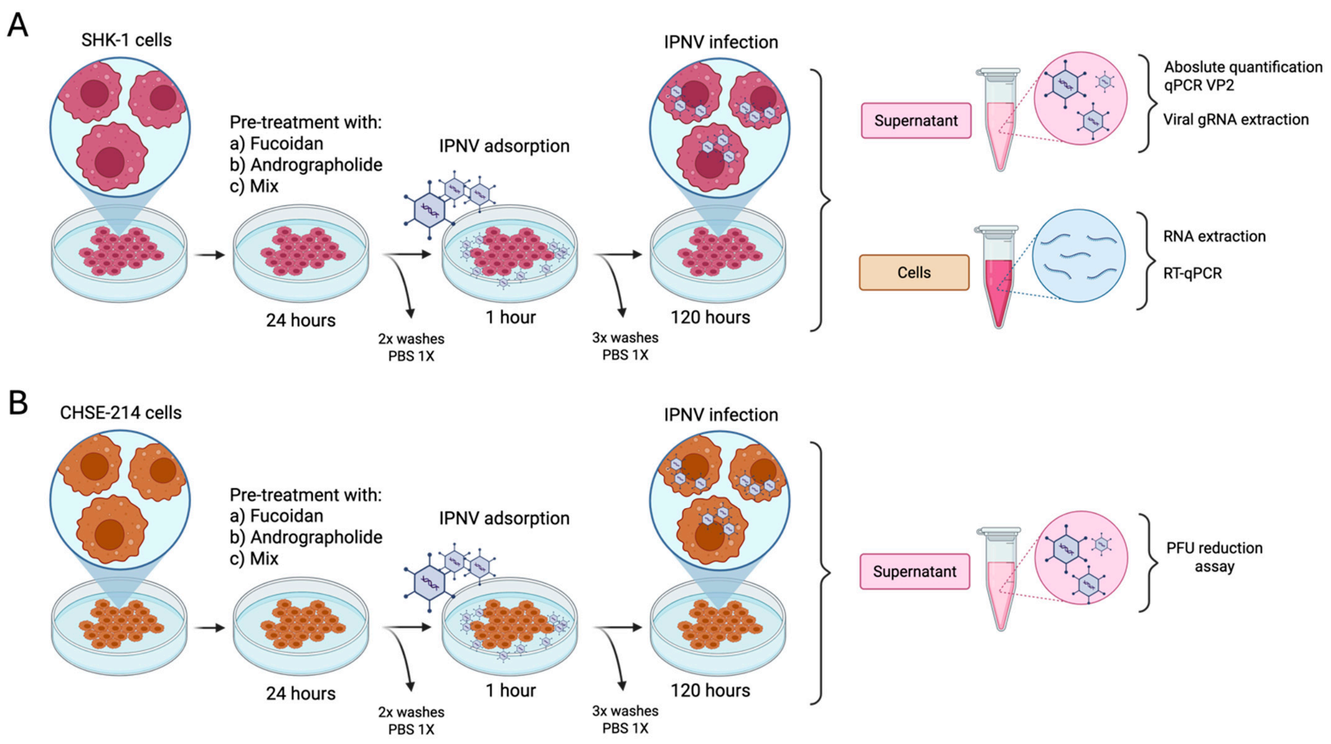

Supplementary Figure S2: Schematic illustration of the experimental design used to evaluate antiviral response in cells pre-treated with andrographolide, fucoidan, and their combination. A) The viral load released from IPNV-infected cells was quantified from the supernatant using absolute quantification via qPCR targeting the VP2 gene. Additionally, transcript expression analysis was conducted on cells to assess the transcript expression of type I IFN and ISGs (PKR, Mx, and viperin). B) The impact of pre-treatment on the production of infectious viral particles in CHSE-214 cells infected by IPNV was evaluated using a PFU-reduction assay. The cartoon illustration was created using BioRender.

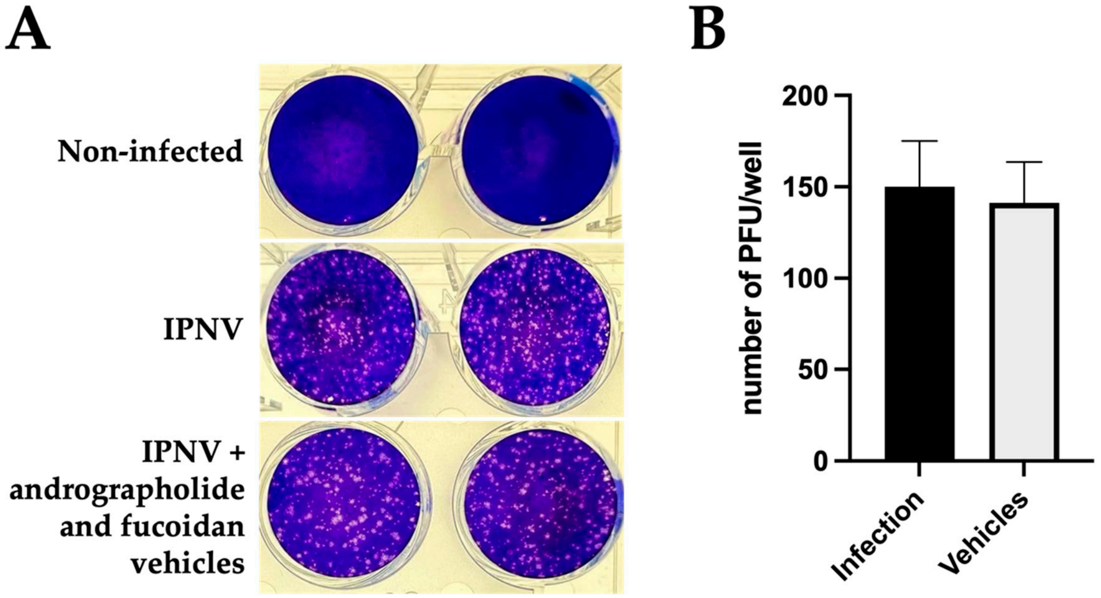

Supplementary Figure S3: The PFU-forming plate assay was visualized on CHSE-214 cells infected with IPNV, which were previously incubated with andrographolide and fucoidan vehicles. A) Representative result from the PFU-forming plate assay **(**the left and right wells represent technical duplicates**).** B) Quantification of PFU/well for each treatment. Statistical analysis was carried out using the non-parametric Mann–Whitney test. Values are presented as the mean ± standard error of the mean from three independent experiments. No statistically significant differences were observed.
